# Episode-like pulse testosterone supplementation induces tumor senescence and growth arrest down-modulating androgen receptor through modulation of p-ERK1/2, pAR^ser81^ and CDK1 signaling: biological implications for men treated with testosterone replacement therapy

**DOI:** 10.18632/oncotarget.22776

**Published:** 2017-11-30

**Authors:** Giovanni Luca Gravina, Francesco Marampon, Patrizia Sanità, Claudio Festuccia, Chiara Forcella, Luca Scarsella, Anna Jitariuc, Antonella Vetuschi, Roberta Sferra, Alessandro Colapietro, Eleonora Carosa, Susanna Dolci, Andrea Lenzi, Emmanuele A. Jannini

**Affiliations:** ^1^ Department of Biotechnological and Applied Clinical Sciences, Laboratory of Prostate Onco-pathology and Experimental Endocrinology, University of L'Aquila, 67100 L'Aquila, Italy; ^2^ Department of Experimental Medicine, Chair of Endocrinology, Sapienza University of Rome, 00161 Rome, Italy; ^3^ Department of Systems Medicine, Chair of Endocrinology and Medical Sexology (ENDOSEX), Tor Vergata University of Rome, 00133 Rome, Italy; ^4^ Department of Biomedicine and Prevention, Section of Anatomy, Tor Vergata University of Rome, 00133 Rome, Italy; ^5^ Department of Biotechnological and Applied Clinical Sciences, University of L'Aquila, 67100 L'Aquila, Italy

**Keywords:** testosterone, prostate cancer, androgens, hypogonadism, pulse treatment

## Abstract

Despite the growing body of knowledge showing that testosterone (T) may not significantly affect tumor progression in hypogonadal patients treated for prostate cancer (Pca), the use of this hormone in this population still remains controversial. The effects of continuous or pulsed T stimulation were tested *in vitro* and *in vivo* on androgen-sensitive Pca cell lines in order to assess the differential biological properties of these two treatment modalities. Pulsed T treatment resulted in a greater inhibition than continuous T supplementation of tumor growth *in vitro* and *in vivo*. The effects of pulsed T treatment on tumor growth inhibition, G0/G1 cell cycle arrest, and tumor senescence was more pronounced than those obtained upon continuous T treatments. Mechanistic studies revealed that G0/G1 arrest and tumor senescence upon pulsed T treatment were associated with a marked decrease in cyclin D1, c-Myc and SKp2, CDK4 and p-Rb levels and upregulation of p27 and p-ERK1/2. Pulsed, but not continuous, T supplementation decreased the expression levels of AR, p-AR^ser81^ and CDK1 in both cellular models. The *in vitro* results were confirmed in an *in vivo* xenografts, providing evidence of a greater inhibitory activity of pulsed supraphysiological T supplementation than continuous treatment, both in terms of tumor volume and decreased AR, p-AR^ser81^, PSA and CDK1 staining. The rapid cycling from hypogonadal to physiological or supra-physiological T intraprostatic concentrations results in cytostatic and senescence effects in preclinical models of androgen-sensitive Pca. Our preclinical evidence provides relevant new insights in the biology of Pca response to pulsed T supplementation.

## INTRODUCTION

The number of subjects requiring Testosterone Replacement Therapy (TRT) secondary to aging or to pathological conditions is dramatically increasing [1]. However, a number of controversial aspects of TRT are currently under debate [2, 3]. One of them is the possibility to use the TRT in deeply hypogonadal patients successfully treated for prostate cancer (Pca). Despite a growing body of knowledge showing that testosterone (T) may not significantly affect tumor progression, the use of T in this clinical condition still remains controversial [2, 3]. A number of studies have investigated the relationship between endogenous T levels and the risk of Pca, but have failed to find a significant relationship [4–7]. This evidence was also supported by a large meta-analysis, which suggested no association between serum androgen levels and the risk of Pca development [8]. Current literature suggests that TRT can be given to symptomatic hypogonadal patients who have received successful radical prostatectomy or radiotherapy and are considered cured [9]. TRT can be started in the first year after radical prostatectomy and with serum PSA levels less than 1 ng/mL after radiotherapy [10, 11]. Differently, the role of TRT for men with untreated Pca managed by active surveillance remains more controversial [2]. Morgentaler proposed the “saturation theory” to explain why T does not directly affect Pca progression during TRT [9]. According to this theory, Pca growth may become insensitive to changes at physiological androgen levels due to saturation of the androgen receptor (AR) by circulating androgens. In agreement with this hypothesis, the intraprostatic androgen environment was found to be almost unaffected in hypogonadal or healthy men treated with exogenous T at low [12] or physiological doses [13]. However when the effect of increasing doses of exogenous T on the concentrations of intraprostatic androgens was investigated in a randomized double blind placebo controlled trial (RCT), it was evident that the average serum T concentrations during treatment positively correlated with both intraprostatic DHT and T after 12 weeks of treatment [14]. These clinical data may be of interest in the light of biological evidence that suggests that prostate cancers have a biphasic response to tumor cell growth in androgen-sensitive Pca cells [15–18]. Androgen supplementation, in the range of picomolar concentrations, makes Pca cells extremely sensitive to androgens [16, 18]. Paradoxically, when tumor cells were exposed to T in the range of nanomolar concentrations, in line with those measured within the prostate when serum T approaches to supraphysiological levels [14] a decrease in cell proliferation was observed [19–21]. So, any treatment with exogenous T administration able to stably or transiently cause supraphysiological serum T concentrations may also increase the intraprostatic T concentrations in the range of inhibitory growth effect. The use of transdermal T gel or of other forms of systemic T delivery allow to obtain stable circulating androgen levels at the higher end of the normal range for T, where many clinicians target therapies for hypogonadal men. Differently, episodic pulsed TRT may be an attractive therapeutic option with respect to the traditional continuous TRT especially if supraphysiological serum T concentrations have to be reached. However, the clinical efficacy of this type of treatment as well as its impact on Pca biology still remain to be studied. Our preclinical study was specifically built to investigate whether the rapid cycling from intraprostatic T concentrations typical of hypogonadism to physiological or supra-physiological concentrations may affect some biological parameters associated with aggressive behavioral properties of preclinical models of prostate cancer.

## RESULTS

### Pulsed testosterone supplementation results in higher decrease of prostate cancer cell growth rate with respect to continuous T treatment

**Figure 1:**
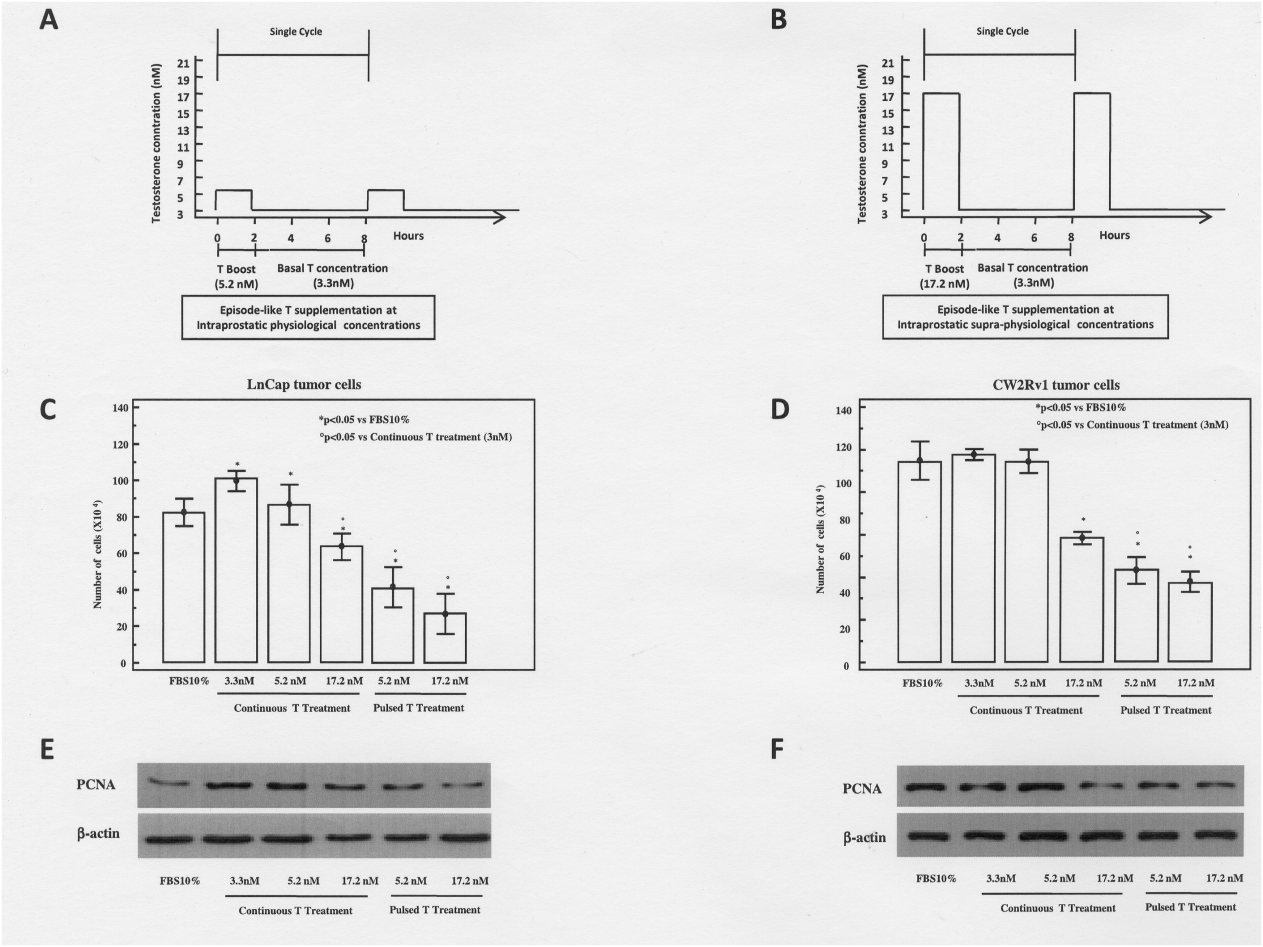
Schematic representation of pulsed T supplementation at physiological **(A)** or supraphysiological **(B)** T concentrations. In the pulsed experiments tumor cells were routinely grown in regular medium supplemented with 10% FBS and T at the concentration of 3 nM. In a time frame of 48 hours these cells underwent 6 cycles of T pulsing. Each cycle lasted for 8 hours and was arranged as follows: T concentration was boosted from 3.3 nM to 5.2 nM (panel A) and to 17.2 nM (panel B) for 2 hours. Then, T concentration was reduced to 3 nM and maintained at this concentration for the remaining 6 hours. Growth at 48 hours of LnCaP **(C)** and CW22rv1 **(D)** cells treated with continuous or pulsed Testosterone The bars show the mean and SD of 36 replicates. Student-Newman-Keuls test for all pairwise comparisons in CW22rv1 and LnCaP cells at 48 hours after treatment. Expression levels, by western blot analysis, of PCNA in LnCaP **(E)** and CW22rv1 **(F)** tumor cells tumor cells treated with continuous or pulsed Testosterone for 48 hours.

It has been previously shown that continuous T treatment exerts a biphasic effect on the proliferative responses of prostatic cancer cell lines *in vitro*, inducing proliferation at concentrations corresponding to those in the cultured medium supplemented with 10% FBS, and inhibition at higher concentrations [15]. For this purpose, tumor cells were treated under both continuous or episode-like T treatments. In Figure 1A and 1B we report the schematic representation of episode-like treatment protocol. In agreement with these data we found that LNCaP and 22rv1 cells, continuously treated with 3.3, 5.2 and 17.2 nM T for 48 h were growth inhibited at the highest T concentration (17.2 nM) (Figure 1C-1D). To investigate whether pulsed T treatment could affect the growth of androgen sensitive Pca cells, cultures were treated for 48 h with pulsed T (5.3 nM or 17.2 nM). Pulsed T treatment resulted in a significant growth inhibition both in LNCaP (67% and 75% at the concentration 5.3 nM and 17.3 nM, respectively) and CW22Rv1 cells (52% and 56% at the concentration 5.3 nM and 17.2 nM, respectively) with respect to mock pulsed control cells. Interestingly, the pulsed T treatment resulted in a significant growth inhibition with respect to continuous T treatment concentrations that was also evidenced by the decrease of PCNA levels only in the pulsed T treatments both in LNCaP and in CW22RV1 cells (Figure 1E-1F).

### T treatment does not induce apoptosis and autophagy in Pca cells

**Figure 2:**
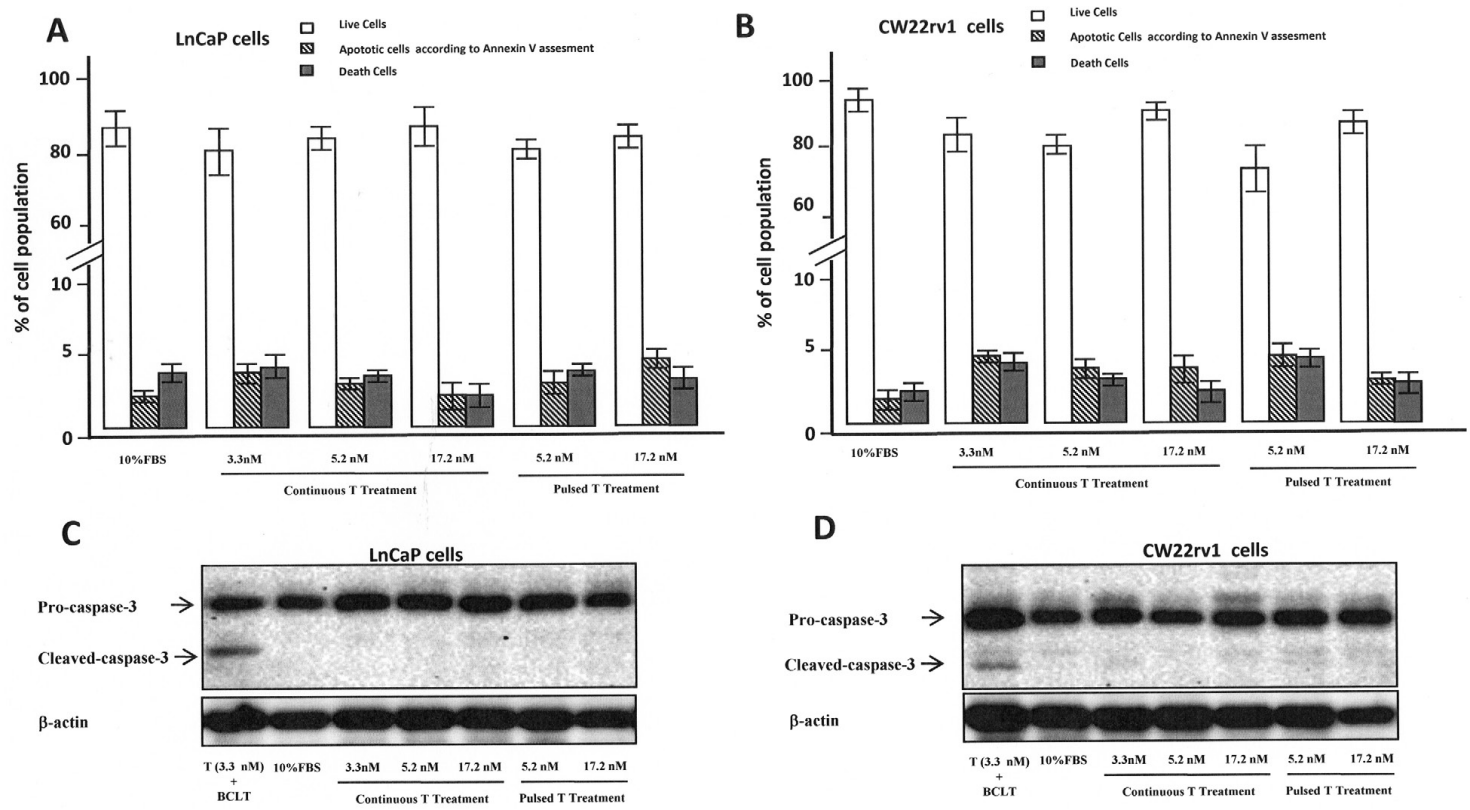
Percentage of live (Annexin V negative/PI negative cells), apoptotic (AnnexinV positive/PI negative cells) and dead (AnnexinV positive/PI positive cells or AnnexinV negative/PI positive cells) cells in LnCaP **(A)** and CW22rv1 **(B)** cell populations treated with continuous or pulsed Testosterone for 48 hours. The bars show the mean and SD of 36 replicates. Student-Newman-Keuls test for all pairwise comparisons in CW22rv1 and LnCaP cells at 48 hours after treatments. Expression levels, by western blot analysis, of pro-caspase-3 and cleaved caspase3 in LnCaP **(C)** and CW22rv1 **(D)** tumor cells tumor cells treated with continuous or pulsed Testosterone for 48 hours.

**Figure 3:**
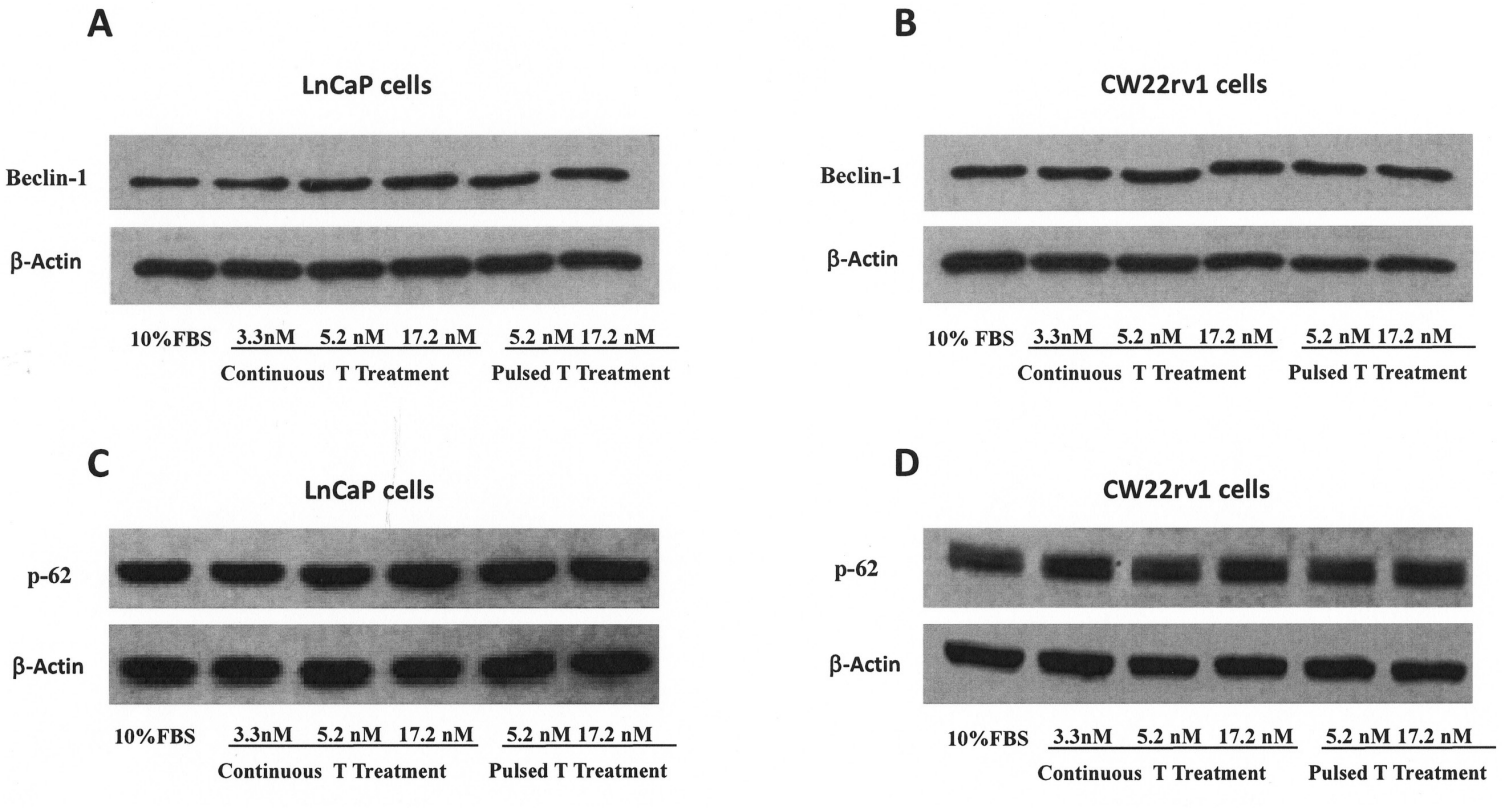
Autophagy assessment Expression levels, by western blot analysis, of beclin-1 **(A, B)** and p-62 **(C, D)** in CW22rv1 and LnCaP tumor cells treated with continuous or pulsed Testosterone for 48 hours.

To understand if the anti-proliferative effect of pulsed T treatment was due to PCa cell death, cells were stained with the Annexin V–Alexa Fluor® 488conjugate and Propidium iodide (PI) (Figure 2A-2B). We found that the percentage of Annexin V positive tumor cells undergoing apoptosis was not influenced by treatment with pulsed or continuous T treatment when compared to cell grown in 10% FBS (Figure 2A-2B), as well as cleaved caspase-3 levels were not influenced by T treatments (Figure 2C-2D). We also checked if autophagy might be induced when tumor cells were cultured with continuous or pulsed T treatments by analyzing the levels of Beclin-1 and p62 [22]. As shown in Figure 3, however, protein levels of these two autophagy markers did not change in T stimulated samples compared to control, suggesting that growth inhibition by pulsed T was not mediated by autophagy.

### Testosterone supplementation induces G0/G1 arrest and tumor senescence

**Figure 4:**
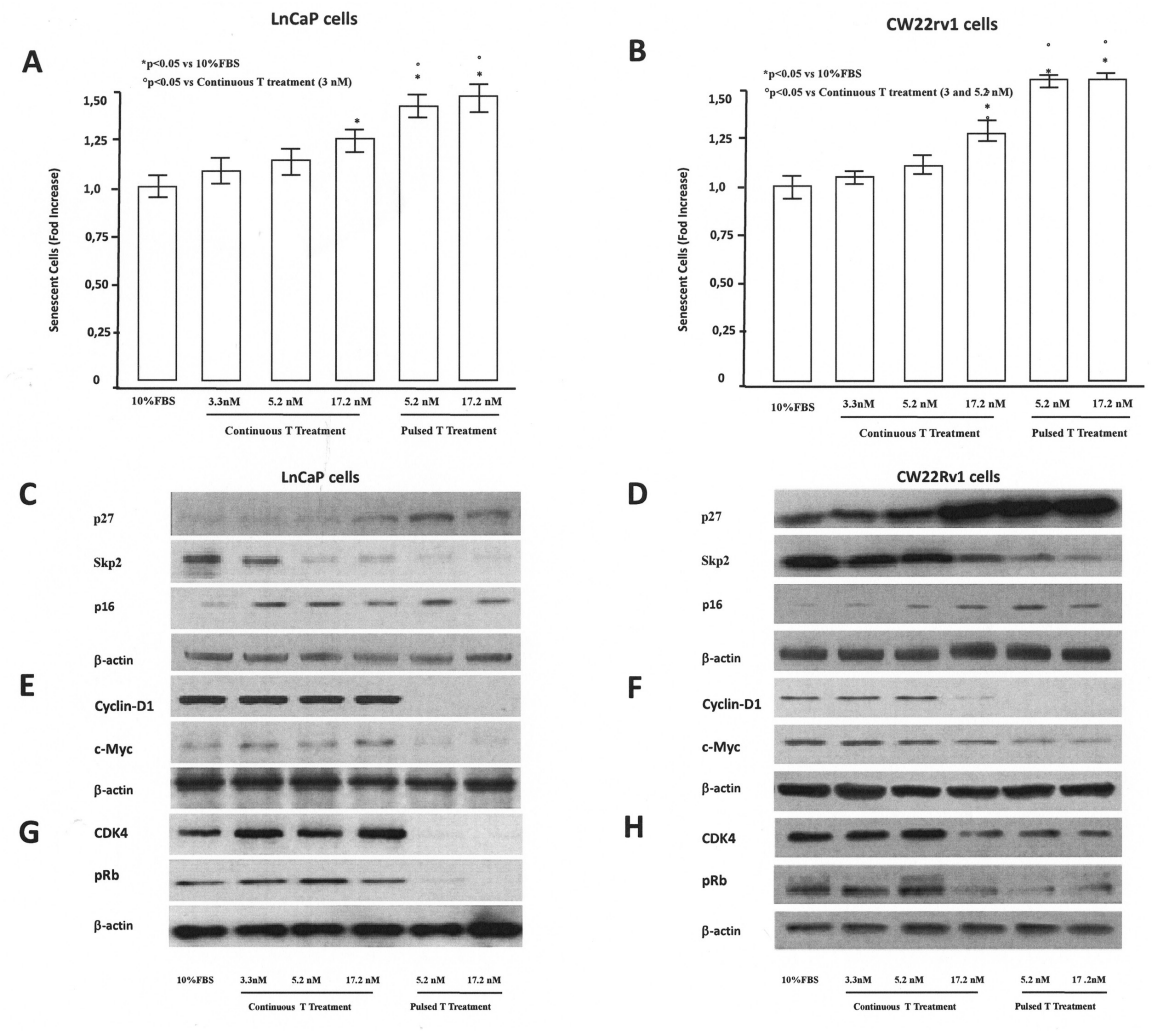
Tumor senescence assessment The content of β–Galactosidase was assessed by a specific ELISA kit in LnCaP **(A)** and CW22rv1 **(B)** tumor cells treated with continuous or pulsed Testosterone for 48 hours. The β–Galactosidase content was expressed as percent versus control (cells cultured in regular medium supplemented with 10% FBS). The bars show the mean and SD of 36 replicates. Student-Newman-Keuls test for all pairwise comparisons in 22rv1 and LnCaP cells at 48 hours after treatments. Expression levels, by western blot analysis, of p27, p16, SKp2 **(C, D)**, cyclin-D1, c-myc **(E, F)**, CDK4 and p-RB **(G, H)** in LnCaP and CW22rv1 tumor cells treated with continuous or pulsed Testosterone for 48 hours.

We next determined whether cell cycle progression of PCa cells was affected by pulsed or continuous T supplementation. LNCaP treated for 48 hrs with pulsated T (5.2 and 17.2 nM) showed an increase of G0/G1 population accompanied by a decrease of cells in both S and G2/M phases (Table 1) with respect to control (10% FBS). Differently, tumor cells, continuously treated with 3.3, 5.2 and 17.2 nM T for 48 h showed and increased G0/G1 population at the highest T concentration (17.2 nM) (Table 1). Also CW22Rv1 tumor cells showed a G0/G1 arrest after pulsed T treatments (5.2 and 17.2 nM), however continuous treatment induced G0/G1 arrest only at higher T concentrations (17.2 nM) (Table 1). Interestingly, in both cellular models, the effect on G0/G1 arrest was more pronounced in the pulsed treatments, without a concentration-dependent effect. Since growth arrest in the G0/G1 phase of the cell cycle can be associated to cellular senescence [24], we studied the effects of T treatments in inducing senescence in LNCaP and CW22rv1 cells. Activity of β-galactosidase, an enzyme that marks the senescence process [20], was thus assessed during T treatments. In LNCaP tumor cells, both continuous and pulsed T treatments induced cellular senescence, as shown by the increase in β-galactosidase activity with respect to control untreated cells (Figure 4A), while CW22Rv1 cellular senescence was induced only by continuous high T (17.2 nM) treatment and by pulsed T treatments (5.2 and 17.2 nM) with respect to the control untreated cells (Figure 4B).

**Table 1:** Cell cycle analysis at 48 hours of treatment

LnCap tumor cells			
Treatment Groups	Phases of cell cycle		
	G0/G1	S	G2/M
**Regular Medium with 10% FBS**	**63%+/-10%**	**22%+/- 6%**	**15%+/- 4%**
***Continuous T treatment (3.3 nM)***	**64%+/-8%**	**16%+/-8%**	**22% +/-6%**
***Continuous T treatment (5.2 nM)***	**69%+/- 4%**	**16%+/- 3%**	**15%+/-8%**
***Continuous T treatment (17.2 nM)***	^*^72%+/-4%	**18%+/- 5%**	**10%+/-2%**
***Pulsed T treatment (5.2 nM)***	^*^89% +/-12%	**6%+/- 3%**	**5%+/-2%**
***Pulsed T treatment (17.2 nM)***	^*^91%+/-8%	**4%+/- 2%**	**5%+/-3%**
^*^P<0.05 vs 10% FBS.			

CW22RV1 tumor cells			
Treatment Groups	Phases of cell cycle		
	G0/G1	S	G2/M
**Regular Medium with 10% FBS**	**58% +/-6%**	**22% +/- 3%**	**20% +/- 5%**
***Continuous T treatment (3.3 nM)***	**60% +/- 8%**	**15% +/-6%**	**23%+/-3%**
***Continuous T treatment (5.2 nM)***	**61% +/- 5%**	**24% +/- 4%**	**16% +/- 5%**
***Continuous T treatment (17.2 nM)***	^*^67% +/-2%	**20% +/- 6%**	**13% +/- 3%**
***Pulsed T treatment (5.2 nM)***	^*^82% +/-10%	**22% +/- 7%**	**5%/3%**
***Pulsed T treatment (17.2 nM)***	^*^83% +/-9%	**14% +/- 4%**	**5% +/-2%**

^*^P<0.05 vs 10% FBS.

**Figure 5:**
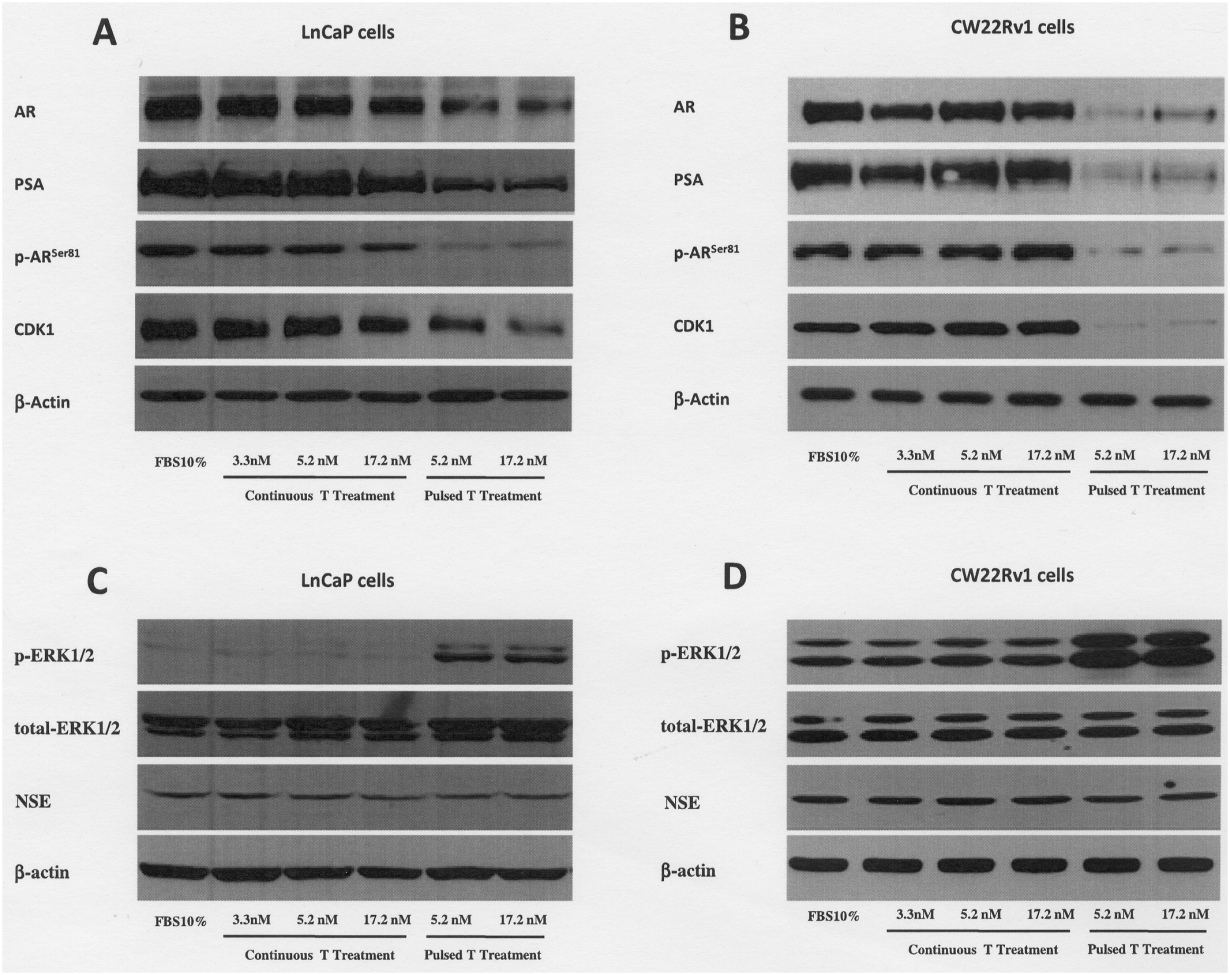
Expression levels, by western blot analysis, of AR, PSA p-AR^ser81^, CDK1, p-ERK1/2, total ERK1/2 and NSE in LnCaP **(A** and **C)** and CW22rv1 **(B** and **D)** tumor cells treated with continuous or pulsed Testosterone for 48 hours.

To explore the molecular mechanism underlying the arrest of tumor cells in G0/G1 phase, we assessed the expression levels of the cell cycle inhibitors p16 and p27, in pulsed or continuous T treatments (Figure 4C-4D). Pulsed, but not continuous, T treatments induced an increase of p16 p27 in LNCaP cells (Figure 5C), while in CW22rv1 cells both pulsed T treatment and continuous T treatment induced a strong increase of p27 and to a lesser extent an increase of p16 levels (Figure 4D). We also found that S-phase kinase-associated protein 2 (Skp2), a protein involved in p27 ubiquitylation and degradation, inversely correlated with p27 both in LNCaP and CW22Rv1 tumor cells. Interestingly, Skp2 levels were down-regulated by continuous or pulsed T treatments both in LNCaP and in CW22Rv1; although in these latter the continuous T treatment was effective only at 17.2 nM T concentration (Figure 4A-4B). We next investigated if T treatment was also affecting cyclin-D levels [28]. By western blot analysis we found that pulsed T stimulation strongly down-regulated cyclin D1 and c-Myc levels both in LNCaP and CW22Rv1 cells. In the latter, similarly to Skp2, only high T concentration (17.2 nM) was also able to reduce cyclin D1 and c-Myc levels compared to the control (Figure 4E-4F). Finally, we evaluated CDK4 and p-RB expression upon pulsed or continuous T treatment. As shown, the levels of these two mediators were strongly down-regulated upon pulsed T treatments both in LNCaP tumor cells (Figure 4G) and in CW22rv1 tumor cells (Figure 4H).

### T treatments results in androgen receptor down-modulation via CDK1 modulation

Since we found that PCa cells were growth inhibited and arrested in G0/G1 of the cell cycle by T treatments, we investigated if AR expression could also be modulated by pulsed or continuous T concentrations. As shown in Figure 5 (panels A-B) we found that pulsed T supplementation induced a sharp decrease of AR protein levels in both cell lines and, as expected, of the AR downstream target prostate-specific antigen (PSA) (Figure 5A-5B). Phosphorylation of AR in Ser-81 is one of the most important mechanisms that increase AR protein stabilization and trans-activation ability [29, 30, 31]. In order to investigate if AR down-regulation might be related to decreased AR stability we probed protein extracts from LNCaP and CW22Rv1 treated with pulsed or continuous T concentrations. We found that both LNCaP and CW22rv1 cells exposed to pulsed T treatment (Figure 5A-5B) showed a marked reduction of p-AR^ser81^ levels, while only continuous treatment with high T (17.2 nM) concentration was able to reduce p-AR^ser81^ levels in CW22rv1 cells. Accordingly, when we evaluated the levels of cyclin-dependent kinase 1 (CDK1), the kinase that phosphorylates Ser81 of AR, we found that pulsed but not continuous T treatment decreased CDK1 expression in both cell lines.

Literature data indicate that high levels of p-ERK1/2 signaling may inversely correlate with AR expression and result in G0/G1 phase cell cycle arrest in LNCaP cells [32]. Additionally, the G0/G1 growth arrest was related to neuroendocrine differentiation, a biological event related to androgens signaling abrogation [32]. When the levels of p-ERK1/2 were assessed upon T treatments, the pulsed treatments increased the phosphorylation levels of ERK1/2 in both cell lines with respect to continuous treatments (Figure 5C-5D). No effect on ERK1/2 phosphorylation levels was observed when tumor cells were exposed to supraphysiological continuous T concentrations with respect to tumor cells cultured in 10% FBS medium (Figure 5C-5D). Interestingly, no changes in NSE levels was found across the continuous and pulsed T treatments. (Figure 5C-5D).

### Growth-inhibitory activity of pulsed supraphysiological T supplementation on a prostate cancer xenograft model in nude mice

**Figure 6:**
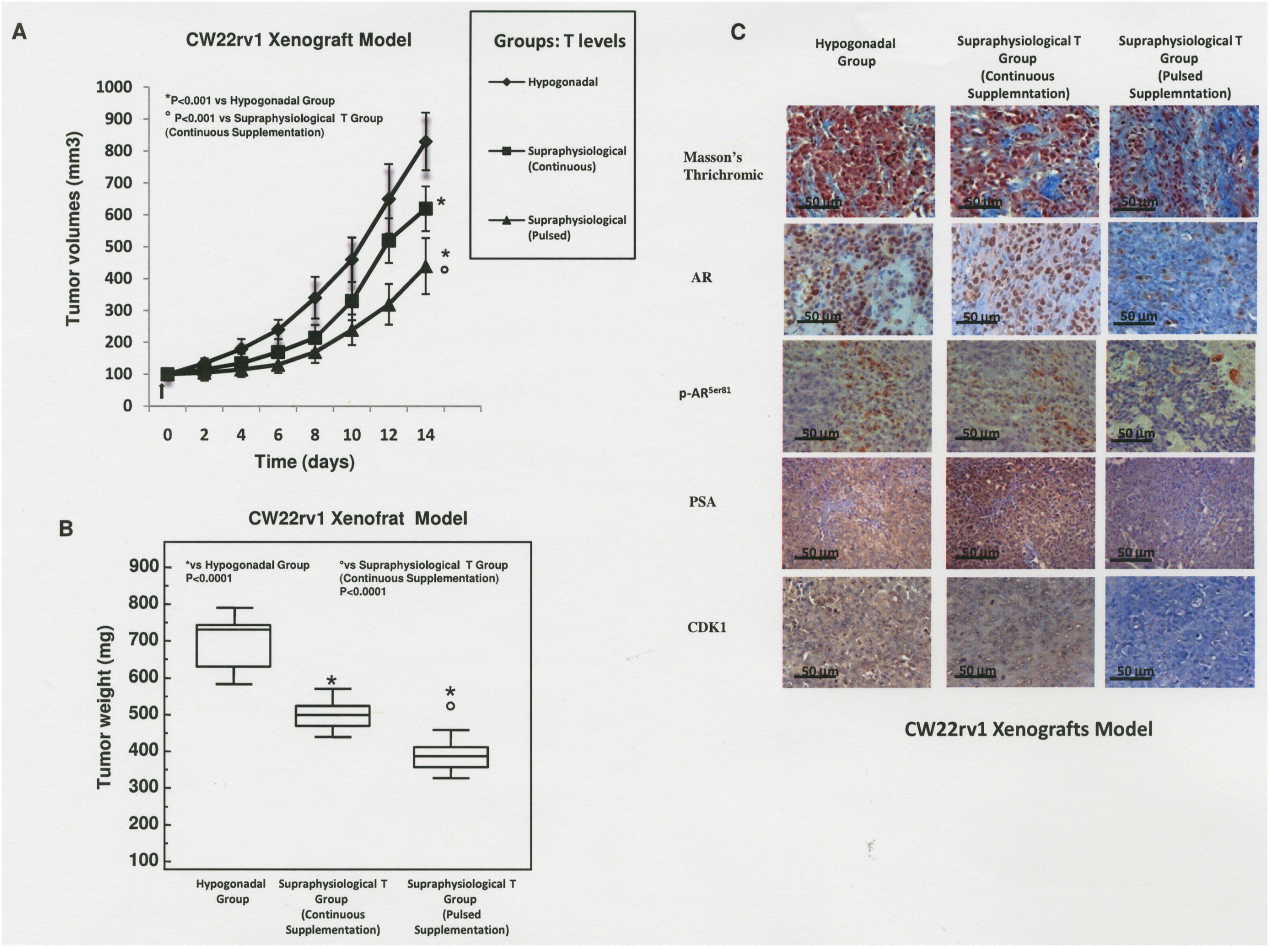
Mice xenografted with CW22rv1 cells were treated with T as described in materials and methods **(A)** Tumor volume was assessed at the beginning of the treatment and every 2 days thereafter. _*_P<0.001 vs Hypogonadal group; ° P<0.001 vs Supraphysiological Continuous T Supplementation **(B)** Tumor weight was assessed at the end of the treatment. ^*^P<0.001 vs Hypogonadal Group; ° P<0.001 vs Supraphysiological Continuous T Supplementation. **(C)** Masson’s trichrome, AR, PSA, p-AR^ser81^ and CDK1 staining in sections from mice xenografted with CW22Rv1 tumor cells.

We next evaluated if continuous or pulsed supraphysiological T supplementation were effective in reducing PCa proliferation *in vivo* in xenograft models. Although CW22rv1 cells showed a lower sensitivity to continuous T treatment compared to LNCaP, they were equally sensitive to pulsed T treatment, and since they represent a primary prostate cancer-derived cell line, we selected these cells to perform xenografts experiments. CW22rv1 cells were subcutaneously inoculated in 6 weeks-old castrated male immunodeficient CD1-nu/nu mice. Serum total T was measured in all animals from the hypogonadal or continuous treatment group two days after silastic tubes implantations and at the end of experiments (after 14 days) by cardiac puncture. Serum T level (ng/mL; mean ± SD) was higher in the supraphysiological T continuous group (23.2 ± 2.2) than in hypogonadal group (1.92 ± 0.34). Differently, in the supraphysiological pulsed T group, serum T was measured at 2 and 5 hours during the first single cycle of treatment. In this group, serum T level (ng/mL; mean ± SD) was at 31.4 ng/mL (± 3.1) and 2.1 (± 3.1) at 2 and 5 hours post injection confirming the rapid T cycling from supraphysiological to hypogonadal serum T levels. A significant reduction in tumor volume was found after 14 days of treatment in the supraphysiological pulsed T group versus the supraphysiological continuous T or hypogonadal groups, as well as when comparing the continuous supraphysiological T group versus hypogonadal groups (Figure 6A). The decrease of tumor volume was paralleled by a decrease of tumor weight in T-treated mice (Figure 6B) and both the parameters correlated with increased tumor fibrosis as demonstrated by trichromic stain (Figure 6C). By immunohistochemical staining we found that positivity for AR, p-AR^ser81^, PSA and CDK1 was strongly reduced in xenografts exposed to pulsed treatments (Figure 6C right panels), while the expression levels of these markers were unchanged upon continuous T treatment or in hypogonadal condition (Figure 6C left and middle panels).

## DISCUSSION

Testosterone is not the cause of prostate cancer, but it is considered essential for the growth of this tumor. The question whether testosterone replacement therapy is a risk factor for PCa remains however controversial. The serum T level required for maximum androgen receptor binding may be as low as 2–4 nM (60–120 ng/dL) [23]. Thus, above this range of concentrations, T may not affect tumor biology or other cellular androgen receptor-dependent mechanisms, as supported by clinical studies showing that cell proliferation markers do not change when serum T levels increase after exogenous T treatment in comparison to subjects treated with placebo [12]. However, a considerable part of the global scientific community remains skeptical regarding the use of TRT in men suffering from a hypogonadal condition and successfully treated for Pca. These negative attitudes may be justified by the relatively low number of clinical and preclinical studies that specifically dealt with how T affects Pca biology at concentrations mimicking those found during TRT. The main clinical objective of all T formulations used during TRT is to obtain a sustained and continuous serum T level in the range of middle/normal values [3]. On the other hand, some authors have studied the pharmacodynamic and pharmacokinetic parameters of T administered in a pulsed way by the sublingual (SL) route [37–39]. They found that the fluctuation in T during SL administration resembled endogenous episodic secretion, with a potentially favorable safety profile [33–36].

Pulsed T treatment has not been investigated, with only some preclinical reports investigating the influence of single T boost on the biological behavior of Pca cells with different sensitivity to manipulation by androgens [33-34, 40, 41]. The main novelty with respect to the current literature is that Pca cells were exposed to a rapid cycling of T both *in vitro* and *in vivo*. When Pca cells were treated with continuous T at concentrations found within the prostate tissue of men under TRT (5.2 nM) no change in tumor cell proliferation was observed compared to tumor cells grown in regular medium or under T concentrations (3.3 nM) found within the prostate tissue of men suffering from hypogonadism. Moreover, when T was supplemented to tumor cells mimicking physiological intraprostatic concentrations, but in a pulse-like manner, a significant decrease in tumor cell proliferation was observed with respect to tumor cells grown under continuous T treatments at a concentration found within prostate tissue of men suffering from hypogonadism (3.3 nM). Additionally, when both pulsed and continuous T were used at supraphysiological concentrations (17.2 nM), the inhibitory effects on cell proliferation rate were more sustained than those observed during the same treatment modalities at lower T concentrations (3.3 nM and 5.2 nM). Our results are in keeping with other evidence. There are, in fact, a number of studies that indicate that T has a biphasic effect on tumor cell growth in androgen-sensitive Pca cells [15, 40, 41]. Testosterone supplementation, in the range of picomolar concentrations, makes Pca cells extremely sensitive to androgens [15, 40, 41]. Paradoxically, when exogenous T was supplemented in the range of nanomolar concentrations, a decrease in cell proliferation was observed [42–44]. The main consequence of our data is that Pca cells exposed to T concentrations similar to those observed during TRT did not acquire more aggressive biological behaviors and conversely, under pulsed treatment, have been found inhibited in their growth.

Another interesting finding we found is that pulsed treatments resulted in a concentration independent cell growth arrest of LNCaP and CW22rv1 cells with concomitant cell cycle arrest in the G0/G1 phase. These effects were not related with apoptotic events. The dissection of underlying molecular mechanisms showed that G1 arrest by pulsed T treatments involves a decrease in SKp2 coupled with increased p21, p27 and p-ERK1/2. Other mediators clearly involved in the G0/G1 cell cycle arrest under pulsed T treatment were cyclin D1 and c-Myc [45]. In controlled cell growth, cyclin D1 leads to phosphorylation of RB which, in turn, mediates the release of E_2_F [46] with activation of c-Myc and cell proliferation [47]. This evidence suggests that a significant decrease in protein levels of cyclin D1 and c-Myc upon pulsed T treatments is an adjunctive mechanism able to exert an inhibitory effect in both LNCaP and CW22rv1 cells. The implications of T-driven modulation of these mediators may be of particular significance in hormone-sensitive Pca cells because cyclin D1, c-Myc and SKp2 are associated with androgen-stimulated growth.

Another way by which pulsed and continuous T treatments influence the biology of Pca cells is linked to tumor senescence induction. Senescence represents an irreversible form of cell-cycle arrest that can act as an important tumor-suppressive mechanism and the literature indicates that growth arrest in the G0/G1 phase of the cell cycle and tumor senescence may be closely correlated [16]. In this regard, Skp2, a critical component of the Skp2–SCF complex, acts as an E3 ligase to target p27 for ubiquitylation and degradation [25, 26]. Recent studies also suggest that Skp2 inactivation induces tumor senescence via p21 and p27 and that this phenomenon may be partially under androgenic control in Pca [24, 25]. In agreement with these findings, we found that tumor senescence, as measured by the surrogate marker β-galactosidase, was significantly increased in Pca cells treated with pulsed or continuous T supplementation compared to controls. Testosterone-mediated tumor senescence paralleled with decreased SKp2 and increased p21 and p27 expression [27].

Another interesting and new finding of our study concerns the modulation of AR expression which has a central role in PCa biology. AR expression was selectively down-regulated when tumor cells were treated with both pulsed physiological or supraphysiological T concentrations. Conversely the expression of this nuclear receptor was almost unaffected under continuous T supplementation. The AR modulation was paralleled by the modulation of p-AR^ser81^, CDK1 and p-ERK1/2, three mediators involved in stabilization of AR [31, 32]. Studies have shown that CDK1 is a p-AR^Ser-81^ kinase and maintains AR expression via Ser-81 phosphorylation. We speculate that the rapid cycling T levels affects CDK1 and p-AR^ser-81^ expression decreasing the AR protein stability with consequent effects on AR protein expression levels. The reasons why T may differentially affect these mediators under continuous or pulsed treatment modalities remains to be elucidated although the differential increase of p-ERK1/2 upon different T treatment regimens may explain this interesting and new phenomenon. In this regard, literature data indicate that high levels of p-ERK1/2 signaling may inversely correlate with AR expression and result in G0/G1 phase cell cycle arrest in LNCaP cells [32]. Some of these *in vitro* results were confirmed in a xenograft model *in vivo*, providing for the first time the concept of proof that supraphysiological pulsed T supplementation induces a significant reduction in both tumor volume and AR, p-AR^ser81^, PSA and CDK1 staining with respect to supraphysiological continuous T supplementation or T levels in the range of hypogonadism.

Our preclinical evidence may have implications for the future clinical studies in the field of TRT. Physiological intraprostatic T concentrations, achieved by continuous T supplementation, appear to not adversely affect the tumorigenic potential of Pca cells. This finding may suggest that men suffering from hypogonadism and successfully treated with Pca may achieve physiological serum and intraprostatic T concentrations with limited, if any, risks of T-dependent tumor progression. Additionally, when supra-physiological intraprostatic T concentrations were achieved, a significant reduction in the tumorigenic potential of Pca cells was observed. On the other hand, when both physiological or supra-physiological intraprostatic T concentrations were achieved by the pulsed T supplementation, a dramatic decrease in some tumorigenic properties was observed suggesting that this type of supplementation may decrease the tumorigenic potential of Pca cell more effectively than continuous treatments. However, the real efficacy and safety of the pulsed T treatment regimens in a clinical setting must be defined and proved using well-designed clinical trials.

This study has a number of limitations. Although the cellular models used represent two well known models of androgen dependent (LnCaP cells) and primary aggressive Pca (CW22rv1 cells) we realize that they may have unique biological behaviours and molecular characteristics with limited possibility to generalize our evidence to primary/localized/hormone naïve Pca. Another potential limit is that we can only speculate that supraphysiological serum T concentrations can really result in similar changes in the androgens within human prostate tissue. Two studies provide empirical evidence concerning the androgen levels within prostate tissue in the setting of low or physiological doses of exogenous T supplementation [12, 13]. These studies show that prostatic androgens are not materially changed, at least at the circulating androgen levels achieved in these studies (physiological serum T levels) although a slight increasing trend was observed in the intraprostatic T concentrations [12, 13]. This evidence fits well with the saturation theory, which postulates that Pca growth may become insensitive to changes at serum physiological androgen levels due to saturation of the AR [2, 9]. However, the paradigm of saturation theory is not completely satisfied when serum T concentration approaches the upper limit of the normal range. A very recent RCT investigated the dose-dependent effects of increasing doses of exogenous T on intraprostatic androgens concentrations in human males [14]. In this study it was evident that the average serum T concentrations during dose-dependent treatment positively correlated with both intraprostatic DHT and T after 12 weeks of treatment [14]. Interestingly the correlation of serum T with intraprostatic T disappeared if men with very low or very high serum T levels were excluded from the analysis. This evidence may support our assumption that the T concentrations used in our study may be potentially achievable within prostate in men with supraphysiological serum T levels. However, whether the intraprostatic androgens may be rapidly cycling during a pulsed exogenous T supplementation remains to be proven. In this regard the most interesting evidence we provide is that the rapid cycling of serum T levels obtained during pulsed T supplementation may indeed result in a measurable antitumor activity in a human xenograft model of primary aggressive Pca. With these limitations we provide highly relevant new insights, both *in vitro* and *in vivo*, into the biology of tumor response to pulsed T supplementation. The rapid cycling from hypogonadal to physiological and supraphysiological T intraprostatic concentrations results in an antitumor effect in preclinical models of Pca. Whether any of this evidence is clinically relevant remains to be proven.

## MATERIALS AND METHODS

### Reagents and tumor cell lines

All of the materials for tissue culture were purchased from Hyclone (Cramlington,). Plasticware was obtained from Nunc (Roskilde, Denmark). Anti-cyclin D1, anti-PCNA, anti-β-actin, anti-pro-caspsase-3, anti-cleaved caspase-3, anti-SKp2, anti-p27, anti-AR, anti-p-ERK1/2, anti-total ERK1/2, anti-NSE, anti-beclin-1, anti-CDK4 and p62 antibodies were purchased from SantaCruz (SantaCruz, CA). Anti-c-Myc and anti-pAR^ser-81^ was purchased from Cell Signaling and Merk Millipore (Billerica, MA, USA). Anti-Rb (phospho S780) (ab47763) antibody was purchased from Abcam. Testosterone was purchased from Sigma-Aldrich (Italian distributor, Milan, Italy) and dissolved and stocked in 100% methanol. MTT was purchased from Sigma-Aldrich (Italian distributor, Milan, Italy). LNCaP and CWR22Rv1 prostate cancer cell line was obtained from the American Type Culture Collection (Manassas, VA, USA). Cells were cultured in Dulbecco's Modified Eagle's medium (DMEM) or Roswell Park Memorial Institute (RPMI) 1640 supplemented with glutamine, pyruvate, gentamycin (Life Technologies) and 10% heat-inactivated fetal bovine serum (10% FBS) (JRH Biosciences; Lenexa, Kansas, United States) and transferred to medium 10% charcoal strip serum (CSS) before T treatment. To minimize the risk of working with misidentified and/or contaminated cell lines, we later stocked the cells used in this report at very low passages and used at <20 subcultures. Periodically, DNA profiling using the Gene Print 10 System (Promega Corporation, Madison, WI) was carried out to authenticate cell cultures, comparing the DNA profile of our cell cultures with those found in GenBank.

### *In vitro* T treatments

Studies estimate that intraprostatic T concentrations in men suffering from hypogonadal conditions are, on average, 3.3 nM while the concentrations of this hormone during TRT treatment are 5.2 nM [12]. In contrast, no empirical evidence exists regarding the intraprostatic concentrations in men with supra-physiological serum T concentrations. Therefore, 3-fold higher T concentrations (17.2 nM) with respect to the physiologic concentration of 5.2 nM measured in the study of Marks [12] seems to be reasonable and was chosen as intraprostatic supra-physiological T concentration.

### *In vitro* pulsed T supplementation

The schematic representation of pulsed T supplementation is shown in Figure 1. It was built on pharmacodynamic data obtained in a study using sublingual administration of testosterone 2-hydroxypropyl-β-cyclodextrin inclusion complex. [33, 34]. In this study, after the administration of a single dose, acute pharmacodynamic changes were observed with supraphysiological serum concentration of T and a rapid decline to below the normal range at 2 h. In our experimental setting, the pulsed experiments tumor cells were routinely growth in regular medium supplemented with 10% FBS and T at the concentration of 3 nM. In at time frame of 48 hours these cells underwent 6 cycles of rapid T cycling arranged as follow: T concentration was boosted from 3.3 nM to 5.2 nM (Figure 1A) or to 17.2 nM (Figure 1B) for 2 hours. Then, T concentration was reduced to 3 nM and maintained at this concentration for the remaining 6 hours. After each modification of T concentration, tumor cells were gently washed by PBS in order to avoid any accumulation of T in the culture medium.

### *In vitro* continuous T supplementation

Treatments were arranged as follows: (i) T was added to medium at the concentration of 3.3 nM and maintained for 48 hours. (ii) T was added to medium at the concentration of 5.2 nM and maintained for 48 hours. (iii) T was added to medium at the concentration of 17.2 nM and maintained for 48 hours. Cells exposed to continuous treatments were handled as cells exposed to pulsed T treatments. Specifically, in order to minimize the risk that some biological effects observed during pulsed T supplementation were imputable to the rapid cycling of other growth factors contained in the 10% FBS or to an external stress due to the replacement of medium or to PBS washing, the culture medium of tumor cells exposed to continuous T concentrations was replaced and washed identically to what was done during the pulsed T supplementation.

### Cell viability measured by MTT assay

Tumor cells were treated with T as previously described. After these treatments, media were removed and 100 μl of MTT was added at the concentration of 0.5 mg/ml; dishes were processed according to the manufacturer’s instructions. Results were expressed as a percentage of vital cells with respect to controls. All experimentations were performed at least three times.

### Annexin-V analysis

To assess apoptosis, cells were treated with continuous or pulsed T in 6-well plates at a density of 1 X 10^5^ cells per well. After incubation for 48 h, cells were harvested and washed three times with PBS. Cells were either fixed with 70% ethanol for cell-cycle analysis or stained with the FITC Annexin V Apoptosis Detection Kit (Life Technologies) for Tali image-based apoptosis assay (Life Technologies). For detection of apoptosis, cells were incubated with Annexin V and Propidium Iodide (PI) for 20 min at room temperature in the dark. Annexin V and PI stained live cells were then loaded on a Tali Cellular Analysis Slide for Tali image-based apoptosis analysis. Cells stained with Annexin and/or PI were counted and each experiment was performed in triplicate.

### Cell cycle analysis

After appropriate treatments, cell viability was analyzed by the Cell Titer 96 assay (Promega, Madison, WI) and adherent cells were trypsinized, pooled with the culture supernatant containing the apoptotic cells already detached from the dish and centrifuged. Cells (1x10^6^) were washed in PBS and fixed for 30 min by the addition of 1 ml of 70% ethanol. After 30 min, the cells were pelleted by centrifugation at 720 g for 5 min, resuspended in 1 ml of DNA staining solution (PBS containing 200 mg/ml RNase A, 20 mg/ml propidium iodide plus 0.1% Triton X-100) and then stained by incubation at room temperature for 60 min. All cells were measured on a FACScan flow cytometer (Becton-Dickinson, Oxford, UK) and analyses using Cell Quest software (Becton-Dickinson). All experimentations were performed in triplicate and repeated at least three times.

### Senescence assay

Senescence was assessed by a specific assay kit measuring β-galactosidase activity in cultured mammalian cells. The β-galactosidase-associated senescence marker was tested as follows: cells were seeded at 5x10^4^ cells per well in 96-well plates and allowed to attach overnight. The following day, tumor cells were treated as described above. Forty-eight hours later, tumor cells were washed twice with phosphate buffered saline (PBS) at pH7.2. β-galactosidase Assay Reagent (100 μl) was added to each well and an incubation for 30 min at 37°C was performed. A β-galactosidase stop solution (100 μl) was added to each well and absorbance was measured at 450 nm using a plate-reading spectrophotometer. The β-galactosidase content was expressed as percent versus control. All experimentations were performed in triplicate and repeated at least three times.

### Western blot analysis

Cells and frozen tissues were washed with cold PBS and immediately lysed with 1 ml of lysis buffer (50 mM HEPES, pH 7.5, 150 mM NaCl, 1% Triton X-100, 1 mM EDTA, 1 mM sodium orthovanadate, 30 mM p-nitrophenyl phosphate, 10 mM sodium pyrophosphate, 1 mM phenylmethylsulfonyl fluoride, 10 μg/ml aprotinin and 10 μg/ml leupeptin). Lysates were electrophoresed in 7% SDS-PAGE, and separated proteins transferred to a nitrocellulose membrane and probed with the appropriate antibodies using the conditions recommended by the antibody suppliers.

### *In vivo* experimental models

Immunodeficient castrated male CD1-nu/nu mice, at 6 weeks of age, were purchased from Charles River (Milano, Italy). Castrated mice were chosen in order to minimize the interference that endogenous T levels could have during the exogenous T supplementation. Before manipulations, all mice were anesthetized with a mixture of ketamine (25 mg/ml) and xylazine (5 mg/ml). CW22rv1 cells were grown to 80% confluence and harvested. Cells were re-suspended in serum free RPMI-1640 medium with penicillin and streptomycin, mixed 1:1 with Growth Factor Reduced (GFR) BD Matrigel Basement Membrane Matrix (BD Biosciences, Palo Alto, California). Using a cold syringe and 27-gauge needle, 5 × 10^6^ CW22rv1 cells were subcutaneously injected into each lateral flank of nude mice. Mice were kept under sterile conditions, receiving sterile nutrition and water. Each group was composed of 10 mice. When measurable tumors (80-130 mm^3^) were established animal treatments were started and were stopped 14 days after. Mice were sacrificed by carbon dioxide inhalation. All the experiments were approved and were carried out in accordance with the relevant guidelines and regulations established by the University of L’Aquila (Medical School and Science and Technology School Board Regulations, complying with the Italian government regulation n.116 January 27 1992 for the use of laboratory animals) which is in line with ARRIVE guidelines.

### Testosterone supplementation *in vivo*

In the continuous T treatment experiments, the group named Supraphysiological continuous T was implanted subcutaneously with 2 mm silastic implants (inside diameter 1.98 mm, outside diameter 3.18 mm, wall thickness 0.61 mm) (Dow Corning Corp. Midland, MI) containing Testosterone propionate (Tp) (Sigma–Aldrich, St Louis, MO, T1875) and sealed with silicone glue (Dow Corning Corp.). These silastic implants were chosen on the basis of the method of calculating release dosage related at the surface area of the T-filled silastic tube [48]. According to this prediction model, the estimation of serum T concentration in the implanted mice should be around the range of supraphysiological T levels [20 ng/ml plasma (69.2 nM)] of an adult (> 40years old) human male. The hypogonadal group, was implanted subcutaneously with 1.0 cm silastic implants (0.078 mm inner diameter, 0.125 mm outer diameter; Dow Corning Corp. Midland, MI) containing Tp (Sigma–Aldrich, St Louis, MO, T1875) and sealed with silicone glue (Dow Corning Corp.). Based on the same method of calculating release dosage based on the surface area of the T-filled silastic tube [48] the estimation of serum T concentrations in the implanted mice should be of around 2 ng/ml plasma (6.9 nM), which is in the range of T levels of hypogonadal adult (> 40years old) human male. In the pulsed T experiments, T (2.5 g) and 2-hydroxipropyl-β-cyclodextrin (25g) were stirred in distilled water (100 ml) at room temperature for 30 minutes, filtered by a Millipore filter (0.45 μm) and then diluted to 200 ml with distilled water and freeze-dried. The resulting powder containing of T was dissolved in isotonic saline solution and injected subcutaneously, as already described [49]. In the pulsed T treatment experiments, T was injected subcutaneously every 8 hours for 14 days. T administered as inclusion complex with 2-hydroxipropyl-β-cyclodextrin subcutaneously enters the circulation in pulsed like manner similar to the sublingual administration of a tablet containing testosterone (150 ng/kg):2-hydroxipropyl-β-cyclodextrin complex to a hypogonadal male [49, 33, 34]. The mice included in this group (supraphysiological pulsed T group) were also implanted with silastic tubes in order to obtain animals with T levels of a hypogonadal adult (> 40years old) human male. The use of silastic implants in this group was necessary in order to avoid that, after single subcutaneous T supplementations, the predicted levels of serum T levels dropped in the range of castration. The silastic tubes were implanted when measurable tumors (0, 8–1, 0 cm^3^) were established and the implants were maintained for 14 days.

### Evaluation of treatment response *in vivo*

The evaluation of treatment response *in vivo* was evaluated as follows: (1) measurement of tumor volume during and at the end of experiments. Tumor volume was assessed by measurement every 2 days with a Vernier caliper (length × width). The volume of the tumor was expressed in mm3 according to the formula 4/3π r3;

### Histological and immunocytochemistry staining

Serial 3 μm sections were stained with Masson’s Trichrome in order to evaluate morphological aspects. For immunohistochemistry (IHC) analysis, sections were incubated for 40 minutes in methanol and 3% hydrogen peroxidase solution and then rinsed in PBS. Samples were incubated 10 minutes in buffered citrate 0.01 M, pH 6, twice and rinsed in PBS. Sections were then treated with BSA (5%) for 10 minutes and finally incubated overnight with specific antibodies against, CDK1 (#ab8040; Abcam, Cambridge, UK), AR (#M3562; Dako UK Ltd.), pAR^S81^ (#07-1375; Merk Millipore, Billerica, MA, USA), and PSA (#sc 7316, SantaCruz, CA) were diluted at 1:200, 1: 100, 1: 1000, 1: 100, respectively. Samples were then rinsed with PBS for 5 minutes and incubated with a labeled streptavidin-biotin- peroxidase conjugate kit (Dako LSAB plus, cod. K0675, Dako Cytomation, Milan, Italy). After rinsing in PBS for 10 minutes the sections were incubated with 3, 3-diaminobenzidine-tetrahydrochloride (DAB, Sigma Aldrich) for 1–3 minutes. The specificity of immune reactions was revealed by the absence of the primary antibodies. Lastly, the samples were counterstained with Mayer’s Hematoxylin and observed under a photomicroscope Olympus BX51 Light Microscope (Olympus, Optical Co. Ltd, Tokyo, Japan). Observations were processed with an image analysis system (IAS, Delta system, Rome, Italy) and were independently performed in a blinded fashion.

### Serum testosterone measurements

Elisa assay of serum testosterone levels were determined by and Enzyme Immunoassay (EIA) Kit for the *in vitro* quantitative determination of testosterone concentration was performed to measure the serum testosterone levels, without serum extraction, after silastic tube implantations. This kit was purchased from BioCheck, Inc. (Foster City, CA) (Cat. BC-1115) its minimum sensitivity for the detection of testosterone was of 0.05 ng/ml.

### Statistics

Continuous variables were summarized as mean and standard deviation (SD). For continuous variables statistical comparisons between control and treated groups were established by carrying out the ANOVA test or by Student *t* test for unpaired data (for two comparisons). When the ANOVA test revealed a statistical difference, pair-wise comparisons were made by Student-Newman-Keuls test. *P* values <0.05 were considered statistically significant. SPSS® version 10.0 was used for statistical analysis.
